# Supplementary material for: Association between maternal pre-delivery body mass index and offspring overweight/obesity at 1 and 2 years of age among residents of a suburb in Taiwan
Source: PeerJ. 2019 Feb 25;7:e6473. doi: 10.7717/peerj.6473 (PMC6394342; doi:10.7717/peerj.6473)
Supplement: Supplemental Information 3 [file peerj-07-6473-s003.docx]

**Supplementary table. Comparison of baseline maternal and offspring demographic characteristics according to pre-delivery maternal body mass index (BMI) between the included and excluded group**

|  | Pre-delivery maternal body mass index | | | | | | | | |
| --- | --- | --- | --- | --- | --- | --- | --- | --- | --- |
|  | Include | Exclude | p | Include | Exclude | p | Include | Exclude | p |
|  | ＜25 kg/m^2^ (n=90) | ＜25 kg/m^2^ (n=20) |  | 25-29.9 kg/m^2^ (n=105) | 25-29.9 kg/m^2^ (n=45) |  | ≧30 kg/m^2^ (n=66) | ≧30 kg/m^2^ (n=19) |  |
| **Maternal demographics** |  |  |  |  |  |  |  |  |  |
| Maternal age |  |  | 0.216 |  |  | 0.780 |  |  | 0.218 |
| ＜ 35 years old | 74 | 14 |  | 77 | 32 |  | 48 | 17 |  |
| ≧ 35 years old | 16 | 6 |  | 28 | 13 |  | 18 | 2 |  |
| Placenta weight | 653.70 ± 151.95 | 631.45 ± 104.46 | 0.736 | 663.23 ± 135.42 | 697.89 ± 129.32 | 0.094 | 654.67 ± 116.97 | 699.21 ± 115.37 | 0.135 |
| Parity |  |  | 0.138 |  |  | 0.853 |  |  | 0.111 |
| Primipara | 48 | 7 |  | 66 | 29 |  | 38 | 7 |  |
| Multipara | 42 | 13 |  | 39 | 16 |  | 28 | 12 |  |
| **Offspring demographics** |  |  |  |  |  |  |  |  |  |
| Gestational age (days) | 39.02 ± 0.92 | 38.97 ± 1.17 | 0.780 | 39.01 ± 0.99 | 39.09 ± 0.96 | 0.785 | 38.81 ± 1.07 | 39.03 ± 1.00 | 0.401 |
| Birth weight (kg) | 3.08 ± 0.25 | 3.13 ± 0.28 | 0.765 | 3.24 ± 0.28 | 3.22 ± 0.30 | 0.481 | 3.22 ± 0.27 | 3.31 ± 0.32 | 0.334 |
| Birth BMI (kg/m^2^) | 12.29 ± 0.90 | 12.44 ± 0.80 | 0.523 | 12.62 ± 1.02 | 12.79 ± 1.00 | 0.407 | 12.76 ± 1.00 | 13.12 ± 1.25 | 0.316 |
| Sex |  |  | 0.087 |  |  | 0.972 |  |  | 0.066 |
| Boy | 46 | 6 |  | 51 | 22 |  | 40 | 7 |  |
| Girl | 44 | 14 |  | 54 | 23 |  | 26 | 12 |  |
| Mode of delivery |  |  | 0.102 |  |  | 0.903 |  |  | 0.502 |
| Vaginal | 80 | 15 |  | 78 | 33 |  | 47 | 12 |  |
| Cesarean section | 10 | 5 |  | 27 | 12 |  | 19 | 7 |  |
| Apgar score |  |  |  |  |  |  |  |  |  |
| 1 minute | 7.97 ± 0.18 | 7.90 ± 0.45 | 0.693 | 7.92 ± 0.49 | 7.96 ± 0.30 | 0.866 | 7.82 ± 0.89 | 8.00 ± 0.00 | 0.528 |
| 5 minutes | 8.98 ± 0.15 | 8.95 ± 0.22 | 0.492 | 8.95 ± 0.35 | 9.00 ± 0.00 | 0.449 | 8.81 ± 0.58 | 9.00 ± 0.00 | 0.528 |

Data are presented as means ± standard deviations or as numbers

**p* values were analyzed using the Mann-Whitney U test (for continuous variables) and Chi-square tests (for categorical variables)
