# Supplementary material for: Association between maternal pre-delivery body mass index and offspring overweight/obesity at 1 and 2 years of age among residents of a suburb in Taiwan
Source: PeerJ. 2019 Feb 25;7:e6473. doi: 10.7717/peerj.6473 (PMC6394342; doi:10.7717/peerj.6473)
Supplement: Supplemental Information 6 — We analyze the additional 103 basal characteristics and find that there is no difference comparing to former 101 basal characteristics data. Therefore, these 103 records can be generalized into our former study population. Basal characteristics of these 103 mother-children pairs was shown in newly added supplementary table. [file peerj-07-6473-s006.docx]

**Supplementary table. Baseline maternal and offspring demographic characteristics according to pre-delivery maternal body mass index (BMI) (The newly added participants)**

|  | BMI | | | *p** |
| --- | --- | --- | --- | --- |
|  | ＜25 kg/m^2^ (n=37) | 25-29.9 kg/m^2^ (n=42) | ≧30 kg/m^2^ (n=24) |  |
| **Maternal demographics** |  |  |  |  |
| Maternal age |  |  |  | 0.345 |
| ＜ 35 years old | 32 (86.5%) | 31 (73.8%) | 18 (75.0%) |  |
| ≧ 35 years old | 5 (13.5%) | 11 (26.2%) | 6 (25.0%) |  |
| Placenta weight | 680.54 ± 176.13 | 656.90 ± 163.98 | 657.00 ± 115.66 | 0.718 |
| Parity |  |  |  | 0.443 |
| Primipara | 20 (54.1%) | 28 (66.7%) | 13 (54.2%) |  |
| Multipara | 17 (45.9%) | 14 (33.3%) | 11 (45.8%) |  |
| **Offspring demographics** |  |  |  |  |
| Gestational age (days) | 39.09 ± 0.82 | 39.06 ± 0.96 | 38.72 ± 1.13 | 0.489 |
| Birth weight (kg) | 3.08 ± 0.26 | 3.20 ± 0.23 | 3.19 ± 0.27 | 0.169 |
| Birth BMI (kg/m^2^) | 12.34 ± 0.72 | 12.56 ± 0.76 | 12.50 ± 0.92 | 0.418 |
| Sex |  |  |  | 0.579 |
| Boy | 15 (40.5%) | 19 (45.2%) | 13 (54.2%) |  |
| Girl | 22 (59.5%) | 23 (54.8%) | 11 (45.8%) |  |
| Mode of delivery |  |  |  | 0.626 |
| Vaginal | 32 (86.5%) | 33 (78.6%) | 19 (79.2%) |  |
| Cesarean section | 5 (13.5%) | 9 (21.4%) | 5 (20.8%) |  |
| Apgar score |  |  |  |  |
| 1 minute | 7.97 ± 0.16 | 8.02 ± 0.15 | 8.00 ± 0.00 | 0.274 |
| 5 minutes | 9.00 ± 0.00 | 9.02 ± 0.15 | 9.00 ± 0.00 | 0.484 |
| Data collected |  |  |  |  |
| 1-year-old (days) | 380.16 ± 25.67 | 377.74 ± 24.50 | 380.21 ± 14.87 | 0.190 |
| 2-year-old (days) | 829.38 ± 34.60 | 813.64 ± 45.81 | 829.88 ± 32.58 | 0.198 |
| 1-year-old body type |  |  |  |  |
| Normal | 35 (94.6 %) | 36 (85.7%) | 22 (91.7%) | 0.399 |
| Overweight + Obesity | 2 (5.4 %) | 6 (14.3%) | 2 (8.3%) |  |
| 2-year-old body type |  |  |  |  |
| Normal | 36 (97.3%) | 39 (92.9%) | 19 (79.2%) | 0.044 |
| Overweight + Obesity | 1 (2.7%) | 3 (7.1%) | 5 (20.8%) |  |

Data are presented as means ± standard deviations or as numbers (proportion)

**p* values were analyzed using the Kruskal-Wallis (for continuous variables) and Chi-square tests (for categorical variables)
